# Analysis of the Expression of *PRDX6* in Patients with Hepatocellular Carcinoma and its Effect on the Phenotype of Hepatocellular Carcinoma Cells

**DOI:** 10.2174/0113892029273682240111052317

**Published:** 2024-01-22

**Authors:** Runhong Mu, Mingzhu Chang, Chuanbo Feng, Yunhe Cui, Tingyu Li, Chang Liu, Yilin Wang, Xiao Guo

**Affiliations:** 1 Basic Medicine College of Beihua University, Jilin, 132000, P.R. China;; 2 School of Pharmacy, Beihua University, Jilin, 132000, P.R. China;; 3 Zhuhai Integrated Traditional Chinese and Western Medicine Hospital, Zhuhai, 519000, China;; 4 Zhuhai Hospital Affiliated to Southern Medical University, Zhuhai, 519000, China

**Keywords:** Hepatocellular carcinoma (HCC), peroxiredoxin 6 (*PRDX6*), lentivirus, gene knockdown, phenotype, RNA sequence data

## Abstract

**Objectives:**

This research aimed to study the expression of *PRDX6* mRNA in hepatocellular carcinoma (HCC) and its effect on the prognosis of HCC. Moreover, the effect of *PRDX6* gene knockdown on the proliferation, migration, and invasion of HepG2 cells mediated by lentivirus was also examined. This study offers a theoretical and experimental basis for further research on the mechanism of PRDX6 in liver cancer and new methods for clinical diagnosis and treatment.

**Methods:**

RNA sequence data of 369 HCC patients were screened through the TCGA database, and the expression and clinical characteristics of *PRDX6* mRNA were analyzed based on high-throughput RNA sequencing data. HepG2 cells were divided into WT, sh-NC and sh-*PRDX6* groups. Real-time PCR and Western blot were used to detect the expression levels of the *PRDX6* gene and protein, respectively. CCK8 method was used to detect the proliferation activity of HepG2 cells, scratch healing test was used to detect the migration ability, Transwell chamber was used to detect the invasion ability, and Western blot was used to detect the expression levels of PI3K/Akt/mTOR signaling pathway and Notch signaling pathway-related proteins.

**Results:**

The expression of *PRDX6* was significantly correlated with the gender, race, clinical stage, histological grade, and survival time of HCC patients (*P* < 0.05). Compared with that in WT and sh-NC groups, the expression level of *PRDX6* protein in HCC patients was significantly lower (*P* < 0.01), the proliferation activity of HCC cells was significantly decreased (*P* < 0.05), and the migration and invasion ability was significantly decreased (*P* < 0.05) in the sh-*PRDX6* group. The expression levels of PI3K, p-Akt, p-mTOR, Notch1, and Hes1 proteins in the sh-*PRDX6* group were significantly lower than those in WT and sh-NC groups (*P* < 0.05).

**Conclusion:**

The expression of *PRDX6* may be closely related to the prognosis of HCC. Lentivirus-mediated *PRDX6* knockdown can inhibit the proliferation, migration and invasion of HCC cells, which may be related to its regulating the PI3K/Akt/mTOR and Notch1 signaling pathways. *PRDX6* is expected to be a new target for the diagnosis and treatment of liver cancer.

## 1 INTRODUCTION

Liver cancer is one of the most common malignant tumors in the digestive system, and its mortality ranks third among all malignant tumors in the world. There are about 250000 new cases and 600000 deaths of liver cancer every year [1]. The survey shows that the incidence of liver cancer in China is significantly higher than that in other countries, and the incidence and mortality of liver cancer in China account for about 50% of those in the world [2]. The treatment methods for liver cancer mainly include hepatectomy, transplantation, local treatment and systematic treatment [3]. However, there are certain risks in operation, and most of them need to be combined with radiotherapy and chemotherapy to control the metastasis of liver cancer cells, and the survival rate of liver cancer patients after treatment is still not satisfactory. At present, some biomarkers, such as serum alpha-fetoprotein, α-fucosidase and dextran-γ-carboxyl thrombin [4-6], have been used for the detection of liver cancer, but still with some problems, such as poor sensitivity and low specificity. Therefore, it is of great significance for the prevention and treatment of liver cancer to find the related molecules that can affect the malignant phenotype of liver cancer cells to explore the molecular mechanism of the occurrence and progression of liver cancer.

The peroxiredoxins (PRDX) family, as an antioxidant protein family, is favored by scientists. It can not only protect cells from the damage and apoptosis mediated by reactive oxygen species but also play an important role in regulating the proliferation and differentiation of cells and the intracellular signal transduction [7]. *PRDX6*, a member of the PRDX family, has dual functional activities of glutathione peroxidase (GPX) and phospholipase A2 [8]. Due to the complexity of *PRDX6* functions, the research on *PRDX6* has been increasing in recent years. Studies have demonstrated that *PRDX6* has a protective effect in different pathological states of the skin, lungs, eyes, gastrointestinal tract, nervous system, and ovary [9-11]. Moreover, it has been reported that *PRDX6* is highly expressed in cancer tissues, such as colon cancer and cervical cancer [12, 13]. It has been found that *PRDX6* plays an important role in the occurrence and progression of many diseases. Currently, the role of *PRDX6* in hepatocellular carcinoma remains elusive.

As the largest cancer gene database at present, TCGA can compare and analyze tumors under different conditions, looking for gene changes related to cancer formation and development [14]. Matching sample analysis by TCGA could provide a scientific basis for clinical diagnosis and treatment of cancer [15]. Therefore, in this study, the expression, prognostic impact and clinical significance of *PRDX6* mRNA in hepatocellular carcinoma (HCC) were analyzed using the TCGA database. Lentiviral transduction is a highly efficient method for genetic modification by integrating exogenous genes into host cells [16]. Therefore, the effects of *PRDX6* on the proliferation, migration, and invasion phenotype of HepG2 cells were investigated by the lentivirus-mediated knockdown of the *PRDX6* gene and its underlying mechanism was explored to provide a theoretical basis for a new targeted diagnosis and treatment strategy of liver cancer.

## 2 MATERIALS AND METHODS

### 2.1 Materials

#### 2.1.1 Data Materials

The clinical and gene expression data of 369 primary liver cancers were downloaded from the TCGA database, and their age, gender, and tumor resection status were statistically analyzed.

#### 2.1.2 Reagents

Human hepatoma HepG2 cells (Center Laboratory, College of Pharmacy, Beihua University, Jilin China), RPMI-1640 (GIBCO, USA), fetal bovine serum (GIBCO, USA), lentivirus and transfection reagent (Shanghai Shenggong Bioengineering Co., Ltd., Shanghai, China), CCK8 Kit (Sigma, USA), trypsin digestive solution (Biyuntian Biotechnology Co., Ltd., Beijing, China), Lipofectamine ^®^ 2000 (Invitrogen, USA), PrimeScriptTMRT kit and SYBR PremixExTaq TMII reagent (Takara, Japan), RIPA lysis buffer (Shanghai Beyotime Biotechnology Co. Lted, Shanghai, China), anti-*PRDX6* monoclonal antibody, anti-Akt polyclonal antibody, anti-pAkt polyclonal antibody, anti-p-mTOR monoclonal antibody, anti-mTOR polyclonal antibody, anti-Notch1 monoclonal antibody and anti-Hes1 monoclonal antibody (Abcam, UK), Electrochemiluminescence reagent (Azure Biosystems, USA), and Transwell chamber (Corning Costa, USA) were used in this study.

### 2.2 Methods

#### 2.2.1 Gene Set Enrichment Analysis

Firstly, the gene set enrichment analysis (GSEA) was performed to generate an ordered gene list based on the correlation between all genes and *PRDX6* expression, and the significant survival difference between high and low *PRDX6* groups was clarified through GSEA, in which 1000 genome alignments were performed for each analysis, the expression level of *PRDX6* was used as the phenotypic label, and normalized enrichment score (NES) was used to rank the enriched pathways in each phenotype.

#### 2.2.2 TCGA RNA Sequencing Data Mining and Statistical Analysis

RNA sequencing data of 80 resected liver tissues and 43 unresected liver tissues were obtained, and the expression of *PRDX6* mRNA and its clinical significance were analyzed. The correlation between the level of *PRDX6* and the survival of patients was confirmed by the Kaplan-Meier method, and the log-rank analysis was carried out to compare the survival curves. The Cox regression model was used for the univariate and multivariate analysis. All analyses were performed using GraphPad prism.

#### 2.2.3 Construction of shRNA Vector Lentivirus

According to the human *PRDX6* mRNA sequence reported in NCBI, the interfering virus and negative control were designed, and the shRNA lentiviral expression vector was constructed by Shanghai Shenggong Bioengineering Co., Ltd. (Shanghai, China). The sequences were as follows: shRNA-F:5’-CCTGGAGCAAGGATATCAATGCTTA-3’;shRNA-R:5’-CCTCGAGAATAGCTATAACGGGTTA-3’. Nucleic acid was constructed into pLKO.1 vector.

#### 2.2.4 Cell Culture and Transfection

HepG2 cells were cultured at 37°C in RPMI-1640 (containing 100 u·mL-1 penicillin and 100 mg·L-1 streptomycin) in a 5% CO_2_ incubator. The logarithmic growth cells were seeded at a density of 8 × 103/well in 96-well plates. The cells were divided into the untransfected control (WT) group, negative control (sh-NC) group and gene knockdown (sh-*PRDX6*) group, with 5 wells in each group. No agent was added to the wells in the WT group, PLKO. One vector was added to those in the sh-NC group and PLKO. One with *PRDX6* shRNA was added to those in the sh-*PRDX6* group. When the transfection rate of cells reached 70-80%, 100 mg·L-1 puromycin was added into the wells for further screening of the lentivirus-transfected cells. After the cells were continued to be cultured for 5-7 days, the cell phenotype test was carried out.

#### 2.2.5 Quantitative Real-time PCR (qRT-PCR)

The *PRDX6* mRNA expression was detected, and the cells were collected. The total RNA was extracted by the Trizol one-step method. The reverse transcription and qRT-PCR were performed using reverse transcription and real- time PCR kits. The following primers were designed with Primer Premier 5.0 and synthesized by Shanghai Biotechnology Co., Ltd. (Shanghai, China): *PRDX6* upstream primer: 5'-TACGGGCCTCCAG-3'; *PRDX6* downstream primer: 5'-GCCAAGCTCTGTG-3'; β-actin upstream primer: 5'-GATGAGATTGGCATGGCTTT-3'; β-actin downstream primer: 5'-CACCTTCACCGTTCCAGTTT-3'. The PCR reaction conditions were 93°C for 10 min, 93°C for 15 s, 58°C for 30 s, and 72°C for 30 s, with 35 cycles. qRT-PCR was performed according to the manufacturer’s instructions, and relative gene expression data were calculated by the Pfaffl method [17].

#### 2.2.6 Detection of the Expression of Proteins by Western Blot

Proteins in the cells lysed by RIPA lysis buffer were extracted. The content of protein in samples was determined by the BCA method, and the protein samples with the same total protein content were prepared. Proteins in the samples were transferred onto PVDF membranes by 12% SDS- PAGE electrophoresis, then 5% skimmed milk powder was used for the blocking on a shaker at room temperature for 2 h. The first antibody diluents (1 : 2 000) were added to the membranes, and the membranes were incubated at 4°C overnight. The membranes were washed 3 times with TBST the next day, 10 min each time. The membranes were incubated with the second antibody at 37°C for 1 h and then washed with TBST in the same way. ECL chemiluminescence solution was used for the development and photographing of the images. The gray values of the bands were analyzed with Image J software. The experiment was repeated three times. The relative protein expression level was calculated according to the following equation:

Relative protein expression level = gray value of experimental group/gray value of corresponding internal reference.

#### 2.2.7 Detection of Cell Proliferation Activity by CCK8 Method

HepG2 and Hep3B cells in each group described above were seeded on 96-well culture plates, with 5 repeated wells in each group, and cultured at 37°C in a 5% CO_2_ incubator. On 0, 24, 48 and 72 hours, after the cells adhered to the wells, 90 μL medium and 10 μL CCK8 were added into the wells, then the cells were incubated at 37°C in a 5% CO_2_ incubator for 2 h, and the OD value of absorbance was detected at 450 nm. The cell experiment in each group was repeated 3 times.

#### 2.2.8 Detection of Cell Migration by Scratch Test

In the scratch test, cells in each group (1 × 106 cells/well) were seeded in 6-well plates. After the transfection for 24 h, a scratch was made on the monolayer of fusion cells with the tip of a 200 μL pipette, in which the pipette tip for scratch was made sure to be vertical, and the cells were continued to be cultured in a serum-free medium. The migration of cells at the different time points (0 and 24 h) was examined under an optical microscope. The experiment was repeated three times, and the wound healing rate was calculated according to the following equation:

Wound healing rate = (scratch interval at 0 h - scratch interval at 48 h)/ scratch interval at 0 h × 100%.

#### 2.2.9 Detection of Cell Invasion by Transwell Assay

Forty-eight hours after transfection, the cells in each group were digested with trypsin, washed twice with PBS, and re-suspended. The cell concentration of the cell suspension was adjusted to 2 × 105 cells/mL, then the cells were seeded in the Transwell chamber, in which 200 μL of the cell suspension was added into the upper chamber and the complete medium was added into the lower chamber, and the cells were cultured for 24 h. Then, the cells were fixed with paraformaldehyde for 20 min, washed with PBS, stained with crystal violet for 15 min, and washed with PBS again, and their color development was observed under an optical microscope after air drying, in which the cells from five fields selected randomly were observed and counted under an optical microscope. The experiment was repeated three times.

#### 2.2.10 Detection of PRDX6 on PI3K/Akt/mTOR Signaling Pathway-related Proteins by Western Blot

The protein samples were electrophoresed by 10% SDS- PAGE, and the proteins were transferred onto PVDF membranes. The membranes were blocked with 5% skim milk at room temperature for 2 h. The primary antibodies were diluted at the ratio of 1:2000 according to the molecular weight of the proteins, and the membranes were incubated with the primary antibodies at 4°C overnight. Then, the membranes were washed 3 times with TBST, 5 min each time, and incubated with the second antibody at the ratio of 1:5000 at room temperature for 2 h. Finally, after washing the membranes, an ECL luminous solution was used to develop them. The experiment was repeated 3 times, and the relative protein expression level was calculated according to the following equation:

Relative protein expression level = gray value of each protein band/gray value of corresponding internal reference.

### 2.3 Statistical Analysis

SPSS19.0 software was used for statistical analysis. The cell proliferation activity and protein expression level in cells were in accordance with normal distribution, and the cell migration rate and the number of invasive cells were expressed as ± s. A *t*-test was used for the comparison between groups, and *P* < 0.05 indicated a value with statistical significance.

## 3 RESULTS

### 3.1 Correlation between the Expression of *PRDX6* and Clinicopathological Features of HCC

**Fig. (1):**
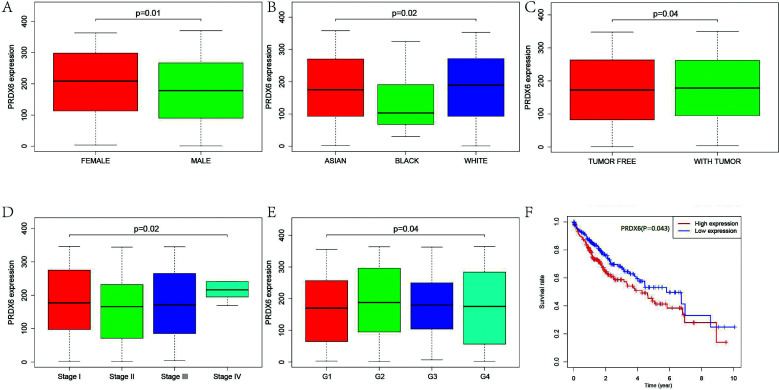
Correlation between the expression of PRDX6 and clinicopathological features of HCC. **(A).** Correlation between the expression level of PRDX6 and the gender of patients; **(B).** Correlation between the expression level of PRDX6 and the race of patients; **(C).** Correlation between the expression level of PRDX6 and the tumor status of patients; **(D).** Correlation between the expression level of PRDX6 and the pathological stage of the patients; **(E).** Correlation between the expression level of PRDX6 and the histological grade of patients; **(F).** Correlation between the expression of PRDX6 and the survival rate of patients.

In the study cohort, there were 250 male research subjects (67.8%), including 175 cases who survived HCC (70.0%), 26 who underwent the tumor resection and died of HCC (10.4%) and 49 who did not undergo the tumor resection and died of HCC (19.6%), and 119 female research subjects, including 71 cases who survived HCC (59.7%), 17 cases who underwent the tumor resection and died of HCC (14.3%) and 31 cases who did not undergo the tumor resection and died of HCC (26.0%). The total survival rate was 66.7% (246 cases), the death rate of cases who underwent the tumor resection was 11.7% (43 cases), and the death rate of cases who did not undergo the tumor resection was 21.6% (80 cases). The correlation between the expression of *PRDX6* mRNA and the clinical pathological features in HCC patients was analyzed. The results showed that the expression level of PRXD6 in males was significantly lower than that in females (*P* = 0.01) (Fig. **1A**), and the expression level of *PRDX6* mRNA in black race was significantly lower than that in yellow or white race (*P* = 0.02) (Fig. **1B**).

The pathological stage of HCC patients can be divided into four stages, and it was found that 132 patients (53.7%) survived in the first stage (Table **1**). The expression level of *PRDX6* mRNA was much higher in patients who underwent the tumor resection (*P* < 0.05) (Fig. **1C**). Compared with that in the other three stages, the expression level of *PRDX6* mRNA in the liver tissue was highest in the fourth stage (Fig. **1D**).

The histopathological grading of HCC included four stages. The results showed that 60.7% of the cases were G1G2, and there were 229 patients in the first and second stages (60.7%) (Table **1**); the expression of *PRDX6* mRNA in G4 was lower than that in G3, G2 and G1 (Fig. **1E**), indicating that the high expression of *PRDX6* has a significant effect on the survival of HCC patients (Fig. **1F**).

### 3.2 Transfection Efficiency of Lentivirus in HepG2 Cells

**Fig. (2):**
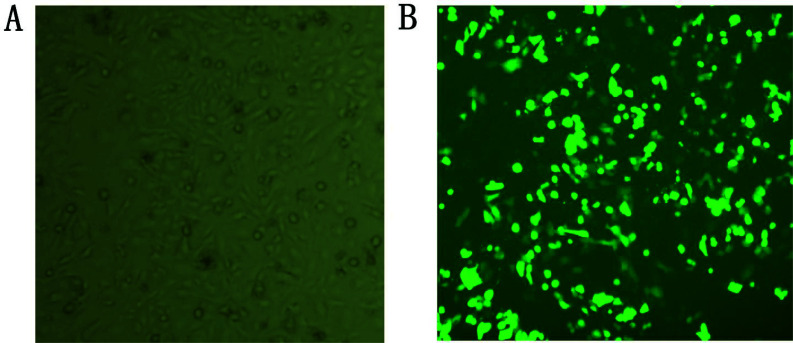
Transfection efficiency of HepG2 cells infected with lentivirus for 5 days under a fluorescence microscope (×100). **(A).** Image of HepG2 cells at normal light. **(B).** Image of HepG2 cells at fluorescence.

The observation results under a fluorescence microscope showed that the infection efficiency of HepG2 cells in the sh-NC group and sh-*PRDX6* group reached more than 80% during the construction of stable lentivirus transfection (Fig. **2**).

### 3.3 Expression Levels of *PRDX6* Gene and Protein in HepG2 Cells

**Fig. (3):**
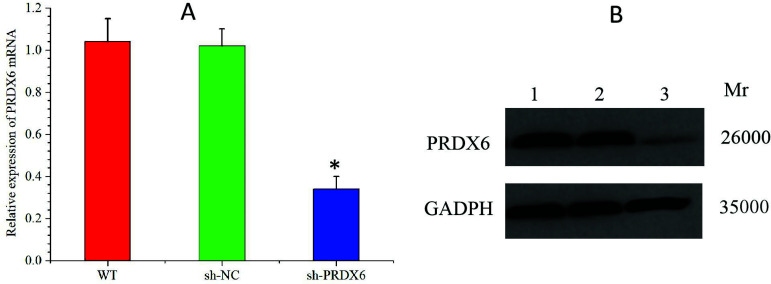
Expression level of PRDX6 mRNA **(A)** and protein **(B)** in HepG2 cells after lentivirus transfection. lane1: WTgroup; lane2: sh-NCgroup; lane3: sh-PRDX6 group.

The qRT-PCR results showed that compared with that in WT group (1.04 ± 0.11) and sh-NC group (1.02 ± 0.08), the expression level of *PRDX6* mRNA in HepG2 cells was significantly decreased in the sh-*PRDX6* group (0.34 ± 0.06). Western blot analysis showed that the expression levels of *PRDX6* protein in HepG2 cells in WT, sh-NC and sh-*PRDX6* groups were 1.02 ± 0.05, 0.96 ± 0.06, and 0.08 ± 0.07, respectively, that is, the expression level of *PRDX6* protein in the sh-*PRDX6* group was significantly lower than that in WT group and sh-NC group (*P* < 0.05) (Fig. **3**).

### 3.4 Wound Healing Rate

As shown in Fig. (**4**), compared with that in the sh-NC group (45.6% ± 5.7%) and WT group (43.1% ± 4.2%), the wound healing rate of HepG2 cells in the sh-*PRDX6* group (34.1% ± 9%) was significantly decreased (*P* < 0. 05) (Table **2**).

### 3.5 Effect of *PRDX6* on the Invasion Ability of HepG2 Cells Detected by Transwell Assay

The invasion ability of HepG2 cells was detected by the Transwell assay. As shown in Fig. (**5**), the invasion ability of HepG2 cells in the sh-*PRDX6* group (53.6 ± 4.8) was significantly lower than that in the sh-NC group (101.9 ± 15.4) and WT group (118.5 ± 8.7) (*P* < 0.01), indicating that the interference of *PRDX6* gene can effectively inhibit the invasion of HepG2 cells. Cells on the lower surface of the filter were visualized and photographed under a microscope, and the relative numbers were counted (five distinct fields per insert).

### 3.6 Effects of *PRDX6* Silencing on the Expression of PI3K/Akt/mTOR and Notch Signaling Pathway-related Proteins

**Fig. (6):**
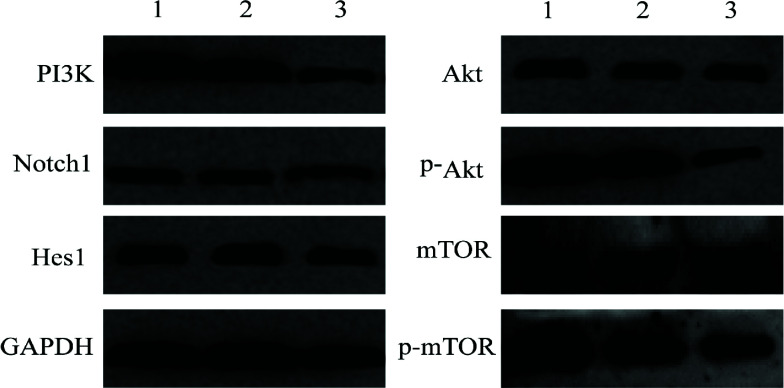
Effects of PRDX6 silencing on the expression of PI3K/Akt/mTOR and Notch signaling pathway-related proteins detected by Western blot. lane1: WT 
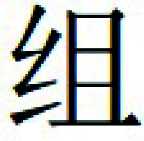
; lane2: sh-NC 
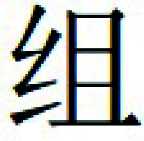
; lane3: sh-PRDX6 
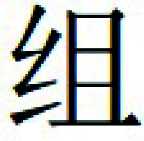
.

Western blot was used to detect the expression of PI3K/Akt/mTOR and Notch signaling pathway-related proteins before and after *PRDX6* knockdown. Compared with those in WT and sh-NC groups, the expression levels of PI3K, p-Akt, p-mTOR, Notch1 and Hes1 proteins in the sh-*PRDX6* group were significantly decreased (*P* < 0.05), and those of the unphosphorylated Akt and mTOR did not significantly change, suggesting that *PRDX6* may participate in the regulation of the PI3K/Akt/mTOR signaling pathway and Notch signaling pathway in HCC (Fig. **6** and Table **3**).

## 4 DISCUSSION

The persistent imbalance of the redox state is one of the important causes of cancer, and the oxidative stress level of cancer cells is usually higher than that of normal cells. Studies have found that PRDX family proteins are up-regulated in many cancers and are almost involved in the whole process of the growth and metastasis of tumors and the regulation of cancer treatment response [18, 19]. Based on its bifunctional enzyme activity, *PRDX6* has more diverse biological functions than other PRDX members. On the one hand, *PRDX6* can scavenge the excessive ROS in cells based on its GPX-like activity; on the other hand, *PRDX6* can protect the cell membrane from the damage caused by oxidative stress based on its PLA2-like activity and then protect the cells. *PRDX6* has been widely studied because of its antioxidant properties at present, and its main function is to protect the body from the damage caused by oxidative stress. It has been reported that *PRDX6* is associated with the occurrence and progression of esophageal cancer [20], ovarian cancer [21], melanoma [22], prostate cancer [23], lung cancer [24], and colorectal cancer [16]. In addition, several studies have reported that high expression of PRDX6 plays an important role in cancer initiation or metastasis [22, 24]. However, the potential effect of PRDX6 on liver cancer remains unclear. Therefore, in this study, the effect of *PRDX6* expression on the survival of patients with liver cancer was analyzed through data mining. It was found that the lentiviral interference could reduce the expression of *PRDX6* in HepG2 cells, and then its effects on the proliferation, migration, and invasion of HepG2 cells were further explored.

Studies have reported that PRDX6 was overexpressed in breast cancer [25]. High levels of PRDX6 expression were detected in samples of tumor tissue from non-small cell lung cancer patients, and expression of PRDX6 presented a positive relationship [26]. It demonstrated that enhanced PRDX6 expression was strongly associated with bladder cancer development [27]. However, there are some limitations in experimental research. Recently, many antioxidant proteins have been found in different kinds of tumors by using bioinformatics analysis, which has become one of the mainstream areas of medical research. Hence, big data analysis is a major challenge in modern biology. Bioinformatics analysis using TCGA high-throughput RNA sequencing data showed that the decreased expression of *PRDX6* was associated with the prognosis in HCC. In addition, we observed that among TCGA HCC cases, aberrant expression of PRDX6 predicts tumor progression and prognosis. In this study, it was found that the mRNA level of *PRDX6* was significantly increased in HCC tissues, which may exclude the factor of insufficient sample size in stage IV. Moreover, the low expression of *PRDX6* in the liver tissue of HCC patients suggests that the prognosis of HCC may be better. *PRDX6* was highly expressed in tissues surrounding the tumor of HCC patients, so it can be considered that *PRDX6*, a member of the PRDX family, may have a unique role in the diagnosis and treatment of liver cancer, but the specific mechanism of its action is still uncertain. A number of limitations of our prognosis analysis based on TCGA data warrant more discussion. Therefore, we further investigated the effect of PRDX6 on the phenotype of HepG2 cells.

Previous studies have shown that *PRDX6* is widely involved in the proliferation, migration, and invasion of a variety of cancer cells. Chang *et al.* [28a] reported that the up-regulation of *PRDX6* can promote the migration of breast cancer cells. He *et al.* [28b] reported that the up-regulation of *PRDX6* could promote the invasion of esophageal cancer cells. On the contrary, the down-regulation of *PRDX6* could reduce the invasion ability of esophageal cancer cells. The proliferation, migration, and invasion of HCC cells is a complex process regulated by many factors, which is also the main reason for the poor prognosis of HCC patients. In this research, the results showed that the knockdown of *PRDX6* could significantly inhibit the proliferation, migration, and invasion of HepG2 cells. In summary, our results confirm the role of PRDX6 in liver cancer development.

PI3K/Akt/mTOR signaling pathway, one of the important pathways of regulating cell cycle, proliferation, apoptosis, differentiation and protein transport, has been a research hotspot of tumor cell biological behavior [29]. A large number of studies have confirmed that the PI3K/Akt/mTOR signaling pathway can accelerate the activity of tumor cells and regulate the receptors to promote the proliferation and metastasis of tumor cells. Notch cell signaling pathway is closely related to the occurrence and progression of tumors. Studies have shown that the overactivity of Notch and the increased expression of Notch1 and Hes1 proteins may indicate a poor prognosis of non-small cell lung cancer [30]. The high expression of Hes1 protein promotes the proliferation of gastric cancer cell lines [31]. Our Western blot results showed that compared to those in WT and sh-NC groups, the expressions of PI3K/Akt/mTOR signaling pathway-related proteins PI3K and Akt were down-regulated, and the proliferation, migration and invasion of HepG2 cells were inhibited in the sh-*PRDX6* group. At the same time, the expression levels of Notch1 and Hes1 were also decreased. Taken together, these findings suggest that *PRDX6* may regulate the development of liver cancer partly through the PI3K/Akt/mTOR signaling pathways. Subsequent studies will be required to elucidate this mechanism further.

## CONCLUSION

In conclusion, the effect of *PRDX6* knockdown on the proliferation, migration and invasion of HepG2 cells was investigated in this study, and the results showed that the knockdown of *PRDX6* could inhibit the proliferation, migration and invasion of HepG2 cells, which may be related to the regulation of PI3K/Akt/mTOR and Notch signaling pathways. The research results may provide a new direction for the in-depth study of the occurrence and progression of HCC and lay a theoretical foundation for the treatment or prevention of liver cancer. Further studies on *PRDX6* gene regulation in human cancers would help us to understand more of its role in cancer development.

## Figures and Tables

**Fig. (4):**
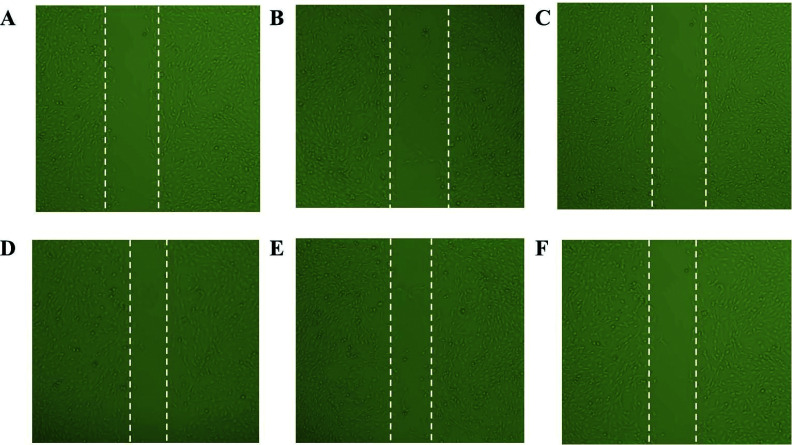
Effect of PRDX6 on the migration distance of HepG2 cells *in vitro*. **(A-C)**: 0 h; **(D-F)**: 48 h; **(A**, **D)**: WT group; **(B**, **E)**: sh-NC group; **(C**, **F)**: sh-PRDX6 group.

**Fig. (5):**
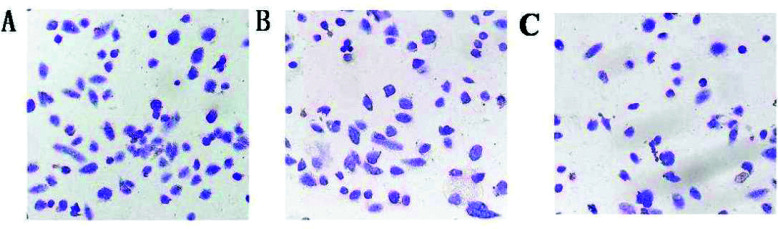
Effect of PRDX6 on the invasion ability of HepG2 cells detected by Transwell assay. **(A)**: WT group; **(B)**: sh-NC group; **(C)**: sh-PRDX6 group.

**Table 1:** Patient characteristics of TCGA cohort.

**-**	**Alive (n=246)**	**Dead with Tumor (n=80)**	**Dead Tumor Free (n=43)**	**Total (n=369)**
Gender	-	-	-	-
Female	71 (28.9%)	31(38.8%)	17 (39.5%)	119 (32.2%)
Male	175 (71.1%)	49(61.3%)	26 (60.5%)	250 (67.8%)
Age	-	-	-	-
Mean(SD)	58.3(13.2)	60.4(13.6)	64.8(13.1)	59.6(13)
Median(min,max)	60(16,84)	60.5(18,90)	67(35,85)	61(16,90)
Pathologic stage	-	-	-	-
Stage I	132(53.7%)	21(26.2%)	19(44.2%)	168(45.5%)
Stage II	61(24.8%)	13(16.2%)	11(25.6%)	82(22.2%)
Stage III	1(0.4%)	1(1.2%)	-	2(0.5%)
Stage IIIA	31(12.6%)	24(30.0%)	10(23.3%)	64(17.3%)
Stage IIIB	5(2.0%)	3(3.8%)	-	8(2.2%)
Stage IIIC	4(1.6%)	3(3.8%)	2(4.7%)	9(2.4%)
Stage IV	1(0.4%)	-	-	1(0.3%)
Stage IVA	1(0.4%)	-	-	-
Stage IVB	-	2(2.5%)	-	2(0.5%)
Histological grade	-	-	-	-
G1	37(15.0%)	11(13.8%)	7(16.3%)	54(14.6%)
G2	119(48.4%)	37(46.2%)	18(41.9%)	170(46.1%)
G3	81(32.9%)	27(33.8%)	14(32.6%)	117(31.7%)
G4	8(3.3%)	2(2.5%)	3(7.0%)	13(3.5%)

**Table 2:** Proliferation activities of HepG2 cells detected by CCK8 assay (*n* = 3,x_ ± s).

**Group**	**Proliferation Activity of HepG2 Cells in Various Groups**				
	**(*t*/h) 4**	**24**	**48**	**72**	**96**
WT sh-NC sh-PRDX6	0.201 ± 0.007	0.636 ± 0.021	0.885 ± 0.021	1.241 ± 0.012	1.521 ± 0.032
	0.194 ± 0.020	0.609 ± 0.007	0.861 ± 0.031	1.279 ± 0.071	1.493 ± 0.028
	0.199 ± 0.005	0.414 ± 0.011^*#^	0.634 ± 0.019^*#^	0.862 ± 0.022^*#^	1.145 ± 0.026

**Note:** *means compared with WT groups *P* < 0.05; ^#^means compared with sh-NC groups *P* < 0.05.

**Table 3:** Expression levels of the PI3K/Akt/mTOR and Notch signaling pathway-related proteins in HepG2 cells (*n* = 3,x_ ± s).

**Group**	**Expression Level of Protein in HepG2 Cells**						
	**Akt**	**p-Akt**	**mTOR**	**p-mTOR**	**PI3K**	**Notch1**	**Hes1**
WT sh-NC sh-PRDX6	0.92 ± 0.04	1.12 ± 0.07	0.93 ± 0.04	1.12 ± 0.07	0.95 ± 0.13	0.87 ± 0.20	1.08 ± 0.07
	0.87 ± 0.02	1.28 ± 0.10	0.89 ± 0.02	1.28 ± 0.10	0.99 ± 0.11	0.82 ± 0.34	1.13 ± 0.02
	0.91 ± 0.03	0.63 ± 0.09^*#^	0.91 ± 0.04	0.63 ± 0.09^*#^	0.78 ± 0.07	0.31 ± 0.06^*#^	0.69 ± 0.12^*#^

**Note:** *means compared with WT groups *P* < 0.05 ;^#^means compared with sh-NC groups *P* < 0.05.

## Data Availability

The authors confirm that the data supporting the findings of this research are available within the article.

## LIST OF ABBREVIATIONS

GPX: Glutathione Peroxidase
GSEA: Gene Set Enrichment Analysis
HCC: Hepatocellular Carcinoma
NES: Normalized Enrichment Score
*PRDX6*: Peroxiredoxin 6
